# Insights into the salinity tolerance of the succulent halophyte *Arthrocnemum macrostachyum*: comparative ecophysiology of plants from heteromorphic seeds

**DOI:** 10.3389/fpls.2024.1504540

**Published:** 2024-12-20

**Authors:** Farah Nisar, Abdul Hameed, Bilquees Gul, Irfan Aziz, Brent L. Nielsen

**Affiliations:** ^1^ Dr. Muhammad Ajmal Khan Institute of Sustainable Halophyte Utilization (MAK-ISHU), University of Karachi, Karachi, Pakistan; ^2^ Faculty of Arts and Sciences, Aga Khan University, Karachi, Pakistan; ^3^ Department of Microbiology and Molecular Biology, Brigham Young University, Provo, UT, United States

**Keywords:** antioxidants, halophytes, heteromorphic seeds, oxidative stress, salinity

## Abstract

**Introduction:**

Little is known about the similarities and differences in responses of plants grown from heteromorphic seeds, which are morpho-physiologically dissimilar seeds produced simultaneously on the same plant.

**Methods:**

In this context, we studied how plants grown from heteromorphic (i.e. black and brown) seeds of the succulent halophyte *Arthrocnemum macrostachyum* respond to increasing salt levels during growth by modulating their physio-chemical processes.

**Results:**

Plants germinated from both black and brown seeds did not show any significant growth reduction and chlorophyll a content decline at moderate salinity (300 mM NaCl) compared to non-saline controls. High salinity (900 mM NaCl), on the other hand, caused decreased growth and sap Ψ_s_ in plants derived from either seed type. In plants emerged from brown but not black seeds, high salinity increased the activity of all H2O2-detoxifying antioxidant enzymes as well as GSH level. Under conditions of high salinity, plants obtained from both types of seeds exhibited signs of lipid peroxidation in the form of elevated malondialdehyde levels.

**Discussion:**

Our findings thus point to metabolic variability in *A. macrostachyum* plants growing from heteromorphic seeds under salt stress.

## 1 Introduction

Coastal marshes, especially of the subtropics, face spatiotemporal fluctuations in sediment salinity, freshwater availability, and seawater inundation (Benfer et al., 2007; Forbes and Dunton, 2006; Sampaio et al., 2021), and thereby are stressful habitats. Marshes are characterized by unique halophyte vegetation with specialized morphological, anatomical, and physiochemical adaptations (Gulzar et al., 2014; Sarika and Zikos, 2021; Zhang et al., 2022; González-Orenga et al., 2024). One of the common adaptations of coastal plants is the development of succulent tissues in leaves and/or stems, which not only store water but also minimize the toxic effects of accumulated salts for long-term survival (Flowers and Colmer, 2008; Zeng et al., 2018; Hameed and Khan, 2011; Grigore and Toma, 2020 and 2021). Succulence is common in a large number of Amaranthaceae, especially species of the subfamily Salicornioideae (Kühn et al., 1993; Tuğ and Başköse, 2023), which are obligate halophytes and require a certain level of salinity for their optimal growth (Flowers and Colmer, 2008). For instance, *Arthrocnemum indicum* (300 mM NaCl; Nisar et al., 2021), *A. macrostachyum* (171-510 mM NaCl; Redondo-Gómez et al., 2010), *Halopeplis perfoliata* (150 mM NaCl; Rasool et al., 2019), *Salicornia europaea* (200–400 mM NaCl; Lv et al., 2012) and *S. dolichostachya* (300 mM NaCl; Katschnig et al., 2013) showed optimal growth under moderately saline conditions. Mechanistically, succulence depends on the efficient compartmentalization of accumulated salts (mainly Na^+^ and Cl^-^) in vacuoles and apoplasts, with concomitant accumulation of compatible solutes in the cytoplasm (Flowers and Colmer, 2008). As a result, succulent tissues possess larger mitochondria to fulfill the excess energy requirements of salt compartmentalization (Nikalje et al., 2018). However, high salinity is inhibitory to the growth of halophytes including highly tolerant Salicornioideae species (English and Colmer, 2013).

Exposure of plants to high salinity also leads to excessive production of reactive oxygen species (ROS; Akyol et al., 2020; Hasanuzzaman et al., 2021), which if accumulated to high levels may cause oxidative damage to proteins, membrane lipids, and nucleic acids (Demidchik, 2015). Halophytes possess a well-coordinated system of enzymatic and non-enzymatic antioxidants to prevent oxidative damage (Surówka M. et al., 2019; Gulzar et al., 2020; Ben Hamed et al., 2020; Hasanuzzaman et al., 2021). Common antioxidant enzymes include superoxide dismutase, catalase, and enzymes of the Foyer-Halliwell-Asada pathway (Ozgur et al., 2013; Gulzar et al., 2020). Ascorbate and glutathione are key non-enzymatic antioxidants, which directly and also in coordination with antioxidant enzymes help plant cells to quench ROS (Jithesh et al., 2006; Hameed and Khan, 2011; Surówka E. et al., 2019). Under low to moderate salinity, antioxidants keep the cellular levels of ROS within a narrow tolerable range (Hameed and Khan, 2011; Ben Hamed et al., 2020). However, under high salinity, the production of ROS often exceeds the capacity of the antioxidant system to detoxify them and thus inflicts oxidative damage to cell components (García-Caparrós et al., 2019; Hasanuzzaman et al., 2021). The salinity threshold inflicting oxidative damage is often >300 mM NaCl in most Salicornioideae halophytes such as *Arthrocnemum indicum* (900 mM NaCl; Nisar et al., 2021), *Salicornia brachiata* (400 mM NaCl; Parida and Jha, 2010), *S. persica* and *S. europaea* (Aghaleh et al., 2011). However, no signs of oxidative membrane damage, measured as MDA accumulation, were evident in *Sarcocornia quinqueflora* even in as high as 1000 mM NaCl salinity (Ahmed et al., 2021). Hence, Salicornioideae halophytes appear to possess an efficient antioxidant defense to deal with salinity-induced ROS production.

Besides physiochemical adaptations, a number of coastal marsh halophytes have evolved the phenomenon of seed heteromorphism as a bet-hedging strategy to survive the heterogeneity of the marsh environment (Liu et al., 2018; Rasheed et al., 2019; Nisar et al., 2019a; Nisar et al., 2019b). Heteromorphic seeds may vary in size (Nisar et al., 2019b) and/or color (Zhang et al., 2021). These differences in seed morphology accompany differential germinability/dormancy and stress tolerance responses of the heteromorphic seeds (Li et al., 2005; 2016; Rasheed et al., 2019). Seed heteromorphism is common in Amaranthaceae including Salicornioideae halophytes. For instance, *Arthrocnemum indicum* (Nisar et al., 2019b), *Salicornia europaea* (Orlovsky et al., 2016), and *S. ramosissima* (Ameixa et al., 2016) produce heteromorphic seeds with two different sizes, while heteromorphic seeds of *A. macrostachyum* differed in color (black and brown; Nisar et al., 2019b). A large number of studies exist that report differences in germination, dormancy, stress tolerance, ecological significance, and physio-chemical attributes of heteromorphic seeds (Liu et al., 2018; Rasheed et al., 2019; Nisar et al., 2019a). However, knowledge about the carryover effects of heteromorphism to later growth stages is scant. For instance, plants derived from heteromorphic seeds of *Suaeda aralocaspica* showed similar growth and physiochemical patterns under both non-saline and saline conditions (Cao et al., 2015). In contrast, plants of *A. indicum* (Nisar et al., 2021) and *Atriplex centralasiatica* (Xu et al., 2011) emerged from heteromorphic seeds showed differences in growth and physio-chemical attributes. Hence, information about the carryover effects of seed heteromorphism on the subsequent growth phase of halophytes appears inconclusive and warrants more studies.

*Arthrocnemum macrostachyum* (Moric) C. Koch (Synonym *Arthrocaulon macrostachyum* (Moric.) Piirainen & G. Kadereit) is a stem-succulent C_3_ perennial euhalophyte of Amaranthaceae (subfamily Salicornioideae), which is commonly found in coastal areas of southern Europe, north Africa, Egypt, Saudi Arabia, Middle East, Iran and Pakistan (Redondo-Gómez et al., 2010; Nisar et al., 2019b; Khan and Qaiser, 2006; ElNaker et al., 2020). It is a densely-branching, erect, glabrous, glaucous-green, succulent, monoecious, perennial, halophyte shrub/sub-shrub with leaves fused to cover nodes, making it apparently leafless (Anonymous, 2024). It is a good source of vitamin E and has high potential to become a gourmet food (Barreira et al., 2017). Its seeds contain about 25% oil with edible quality (Weber et al., 2007; ElNaker et al., 2020). It has been used as an antibiotic (Zabka et al., 2011) and alexipharmic remedy by locals in Tunisia (ElNaker et al., 2020). Extracts of *A. macrostachyum* also have hypoglycemic properties (Sekii et al., 2015; Al-Tohamy et al., 2018). *Arthrocnemum macrostachyum* has a high tolerance to salinity during both germination (600 mM NaCl, Nisar et al., 2019b) and growth stages (1030 mM NaCl; Redondo-Gómez et al., 2010). It produces heteromorphic seeds, which vary in color (black and brown; Nisar et al., 2019b). Germination requirements, stress tolerance, and biochemical responses of heteromorphic seeds of *A. macrostachyum* have been examined (Nisar et al., 2019a, b). However, information about the growth and physiochemical attributes of plants derived from heteromorphic seeds of *A. macrostachyum* is absent. This study aimed to provide answers to the following questions: 1) Do plants obtained from heteromorphic seeds vary in growth response and salinity tolerance? 2) Are there any differences in the osmotic adjustment pattern of plants developed from heteromorphic seeds in response to salinity increments? 3) Do plants derived from heteromorphic seeds vary in their photosynthetic potential under increasing salinity? 4) What are the similarities and/or differences in the redox homeostasis response of plants emerged from *A. macrostachyum* heteromorphic seeds?

## 2 Materials and methods

### 2.1 Plant habitat and seed collection

Seeds of *Arthrocnemum macrostachyum* (Moric) C. Koch were collected from a large population found in a dry coastal-marsh pan adjacent to the Gaddani ship-breaking yard (Latitude: 25° 4’36.62”N; Longitude: 66°42’35.91”E; Distance from seafront: ~300 m) of the Lasbela District, Balochistan, Pakistan. The seed collection site has a hot, dry sub-tropical climate and is dominated by halophyte vegetation. Seeds were scrubbed manually to separate from the inflorescence husk, surface sterilized with 1% (v/v) sodium hypochlorite for 1 minute, rinsed with distilled water, and air-dried. Dimorphic (i.e. black and brown) seeds were then manually separated and stored in clear plastic petri-plates at room temperature (~25-30°C) until use (~6 weeks).

### 2.2 Growth conditions

Heteromorphic seeds were sown separately in shallow plastic trays (7.5 cm depth) containing garden soil and irrigated with water until seedlings reached the two-node stage. When plants were 3 months old the seedlings were transplanted into plastic pots (Size = 11.5 cm in diameter and 25.5 cm in length) filled with sand and sub-irrigated with half-strength Hoagland solution (Epstein, 1972). After 20 days of acclimation, salinity (300 and 900 mM NaCl) was gradually introduced at the rate of 50 mM NaCl after every 12 hr to avoid osmotic shock. Plants irrigated with Hoagland solution served as control (0 mM NaCl). The growth experiment was conducted in a net-house under ambient conditions (average day/night temperature was 37.6/25°C, Photosynthetic photon flux density (PPFD) at midday was ~909.8 μmol m^-2^ s^-1^). There was one plant per pot and there were at least four replicates (n = 4) per treatment. Growth and different physio-chemical parameters were examined after 28 days of NaCl treatments.

### 2.3 Growth parameters

Shoot and root length and fresh weight (FW) was measured immediately after harvest. The dry weight (DW) of plant parts was determined after drying in an oven at 60 °C for 48 hrs. The moisture content of the shoot and root was calculated by using the following formula:


$$\text{Moisture content}\;({\text{g Plant}}^{-1})=\left(\text{FW}-\text{DW}/\text{FW}\right)\times 100$$

The succulence of shoots and water content of roots was determined by using the following formula:


$$\text{Succulence}\;({\text{g H}}_{2}{\text{O g}}^{-1}\text{DW})=\left(\text{FW}-\text{DW}\right)/\text{DW}$$

### 2.4 Sap osmolality

Shoot and root sap osmolality was measured by expressed sap according to Koyro and Huchzermeyer (2004) by using a Dew-point microvoltmeter (Wescor HR-33T, USA). The osmotic potential (Ψ_s_) was calculated using the van’t Hoff equation described by Guerrier (1996):


$$\Psi_{\text{s}}=-\text{nRT}$$

where n is the number of moles of solute, R = 0.008314 J mol^-1^ K^-1^ (gas constant) and T = 298.8 K (absolute temperature).

### 2.5 Photosynthetic pigments

Photosynthetic pigments (Chlorophyll *a, b*, and carotenoid) of freshly collected shoots were extracted with 100% ethanol in tightly capped glass test tubes stored in the dark at 4°C (Ritchie, 2006). Pigment estimation was carried out according to the method of Lichtenthaler and Buschmann (2001) with the help of a UV-Vis Spectrophotometer (Beckman-Coulter DU-730).

### 2.6 Hydrogen peroxide and lipid peroxidation levels

Shoot samples were ground fine with mortar and pestle under liquid nitrogen, homogenized with ice-cold trichloroacetic acid (TCA, 3% w/v), and the homogenate was centrifuged at 12000×g for 20 minutes at 4 °C. The supernatant was used to quantify levels of hydrogen peroxide (H_2_O_2_) according to the method of Loreto and Velikova (2001) and lipid peroxidation according to the method of Heath and Packer (1968).

### 2.7 Enzymatic antioxidants

Shoot tissues were finely ground under liquid nitrogen and homogenized with potassium phosphate buffer (pH 7.0) containing 2% (w/v) polyvinyl polypyrrolidone, 1 mM ascorbic acid, and 5 mM disodium EDTA. The homogenate was centrifuged at 12000×g for 20 minutes at 4°C and the supernatant was used to estimate activities of superoxide dismutase (SOD; EC 1.15.1.1), guaiacol peroxidase (GPX; EC 1.11.1.7) and glutathione reductase (GR; EC 1.6.4.2) using methods described in Hameed et al. (2012). For the extraction of catalase (CAT; EC 1.11.1.6) and ascorbate peroxidase (APX; EC1.11.1.11) finely ground shoot tissues were homogenized in potassium phosphate buffer (pH 7.0) containing 4% (w/v) polyvinyl polypyrrolidone, 1 mM ascorbic acid and 5 mM disodium EDTA and centrifuged at 12000×g for 20 minutes at 4°C. The supernatant was mixed with the same volume of acetone (99.8%) containing 10% TCA and 50 mM dithiothreitol followed by overnight incubation at -20°C. Then the mixture was centrifuged at 12000×g at 4°C for 20 minutes. The collected supernatant was used to estimate the activities of CAT and APX by methods described in Hameed et al. (2012).

### 2.8 Non-enzymatic antioxidants

Shoot TCA extracts were used to quantify ascorbate (AsA) and dehydroascorbate (DHA) by using the method of Law et al. (1983). Reduced (GSH) and oxidized (GSSG) glutathione was quantified according to the method of Anderson (1985).

### 2.9 Statistical analyses

Two-way analysis of variance (ANOVA) was performed to find out whether seed morphology (M), salinity (S), and their interaction (M×S) affected different parameters significantly. A *post hoc* Bonferroni test was used to indicate significant (P<0.05) differences among individual means of the treatments. For all the variables for which the assumption of the homogeneity of variances (Levene’s test) was not met, Welch’s Analysis of variance (ANOVA) was performed. Student *t*-test (P<0.05) was used to compare the responses of plants derived from black and brown seed morphs within each salinity treatment. All statistical analyses were carried out in SPSS version 20.0 for windows (SPSS, 2015).

## 3 Results

### 3.1 Growth parameters

**Figure 1:**
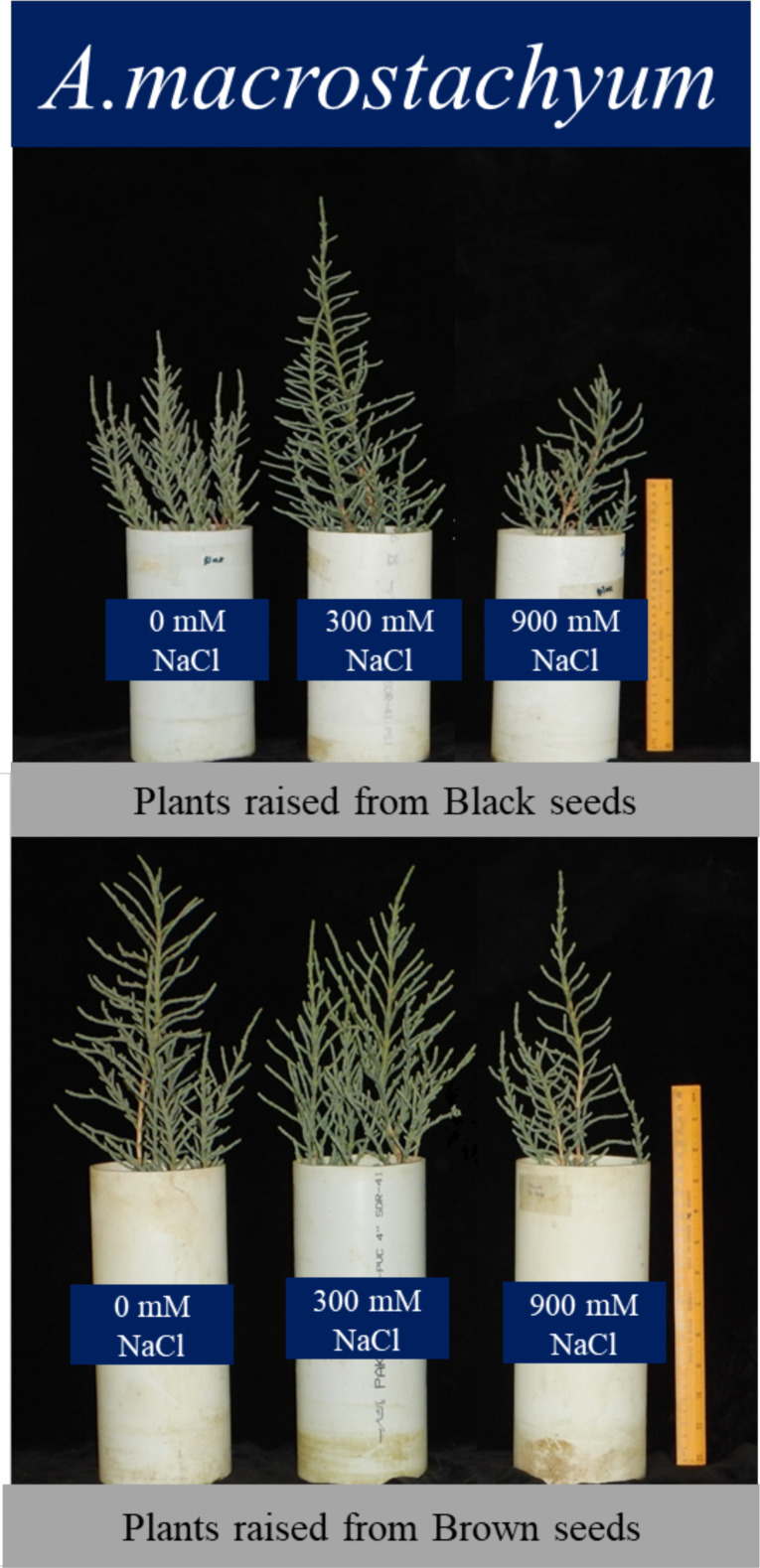
Plants of *A. macrostychum* raised from black and brown seed morphs under different NaCl treatments.

**Figure 2:**
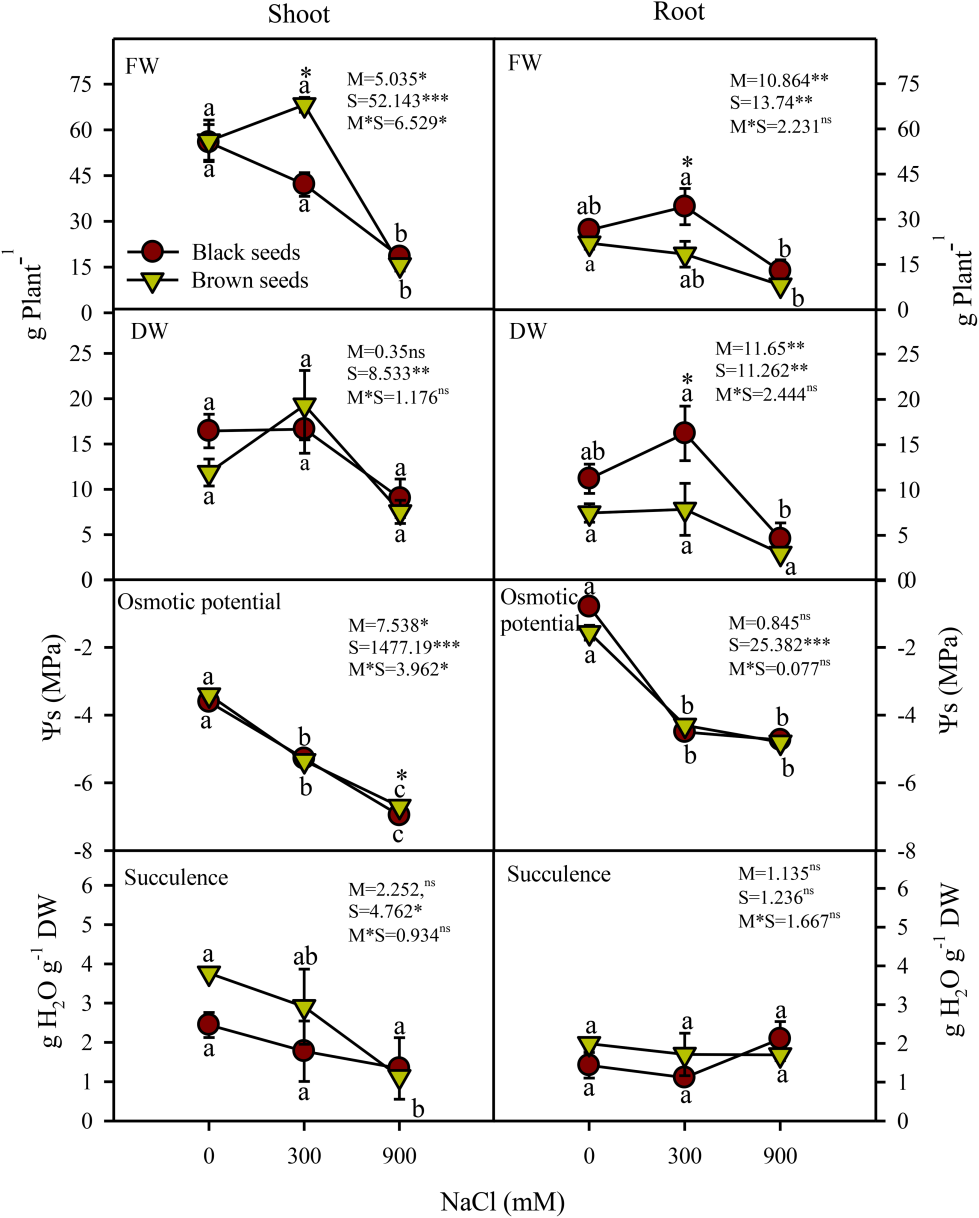
Plant shoot and root height (cm Plant^-1^), fresh weight (FW; g Plant^-1^), dry weight (DW; g Plant^-1^) osmotic potential (MPa), succulence (g H_2_O g^-1^ DW) of plants germinated from black and brown seed morphs of *A. macrostychum* under different NaCl treatments. Data is given as mean ± standard error. Symbols with different letters are significantly (P <0.05) different from each other (Bonferroni test).

*Arthrocnemum macrostachyum* seeds are of two morphologies: black and brown. We investigated the growth responses of the plants developed from heteromorphic seeds under increasing salinity (**Figure 1**). Two-way ANOVA indicated significant (P<0.05) effects of seed morphology (M), salinity (S), and their interactions (M×S) on fresh biomass (FW) of both shoots and roots of *A. macrostachyum* (**Figure 2**). Shoot and root FW of the plants germinated from heteromorphic seeds did not vary from each other under control and high (900 mM NaCl) salinity, but significant (P<0.05) differences were observed under moderate (300 mM NaCl) salinity in FW of plants grown from black and brown seeds (**Figure 2**). Likewise, FW of the plants derived from heteromorphic seeds in moderate salinity was comparable to the control, but a significant (P<0.05) decrease in FW of the plants irrespective of seed origin was observed in high salinity. Root but not shoot dry biomass (DW) varied significantly (P<0.001) between plants grown from heteromorphic seeds. Salinity had a significant (P<0.001) effect on DW of both shoots and roots of plants germinated from either seed type. Similar to FW, the DW of plants obtained from heteromorphic seeds was inhibited only at high but not moderate salinity (**Figure 2**).

### 3.2 Water-related parameters

Shoot osmotic potentials (Ψ_s_) of plants grown from heteromorphic seeds decreased (i.e. became more negative) with increasing salinity, whereas root Ψ_s_ decreased substantially under saline conditions with comparable values in 300 and 900 mM NaCl (**Figure 2**). The Ψ_s_ values of the two types of plants were generally similar and shoots had lower Ψ_s_ than the roots. Root water contents of the two types of plants was comparable and also did not vary with salinity increments (**Figure 2**). Similarly, shoot succulence of plants from black seeds remained unaffected under increasing salinity. Plants from brown seeds had higher shoot succulence compared to those from black seeds in 0 (2 fold) and 300 mM (1.5 fold) NaCl treatments, but a 25% decrease in their shoot succulence occurred in 900 mM NaCl compared to the non-saline control (**Figure 2**).

### 3.3 Photosynthetic pigments

**Figure 3:**
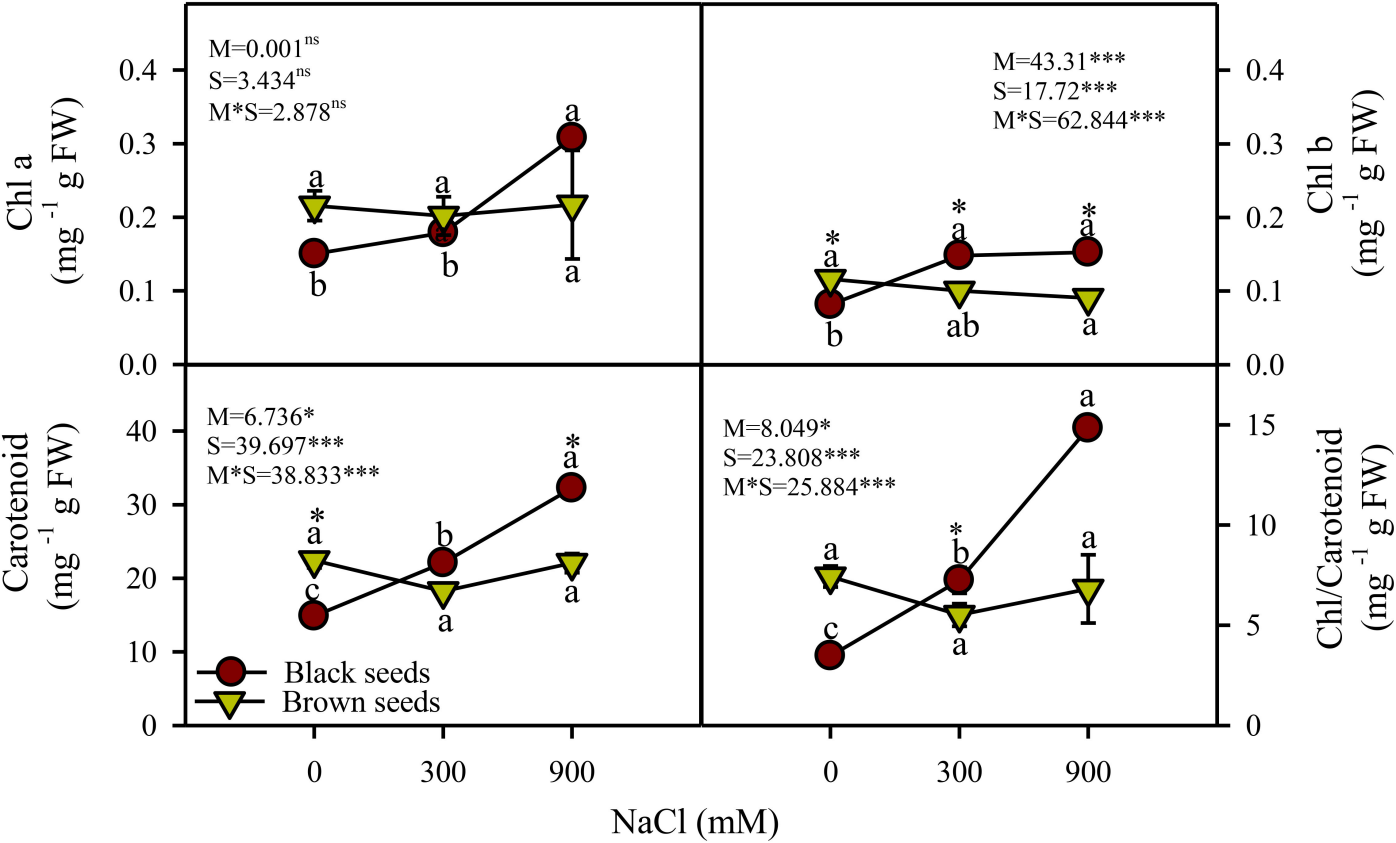
Photosynthetic pigments (mg g^-1^ FW) of plants obtained from black and brown seed morphs of *A. macrostychum* under different NaCl treatments. Data is given as mean ± standard error. Symbols with different letters are significantly (P <0.05) different from each other (Bonferroni test).

Two-way ANOVA indicated a significant (P<0.05) effect of seed morphology (M), salt treatments (S), and their interactions (M×S) on photosynthetic pigments. Chlorophyll *a* (Chl *a*) content of the plants germinated from black seeds in moderate and high salinity treatments were comparable and significantly (P<0.05) higher, respectively, in comparison to the control (**Figure 3**). Chl *a* content of plants grown from brown seeds did not vary across salinity treatments. Chl *b* and carotenoid contents of plants derived from black seeds increased under saline conditions, while those of plants germinated from brown seeds did not vary with salinity (**Figure 3**). Plants obtained from black seeds had higher (~1.5 fold) Chl *b* and carotenoid contents than those from brown seeds, particularly under high salinity.

### 3.4 Hydrogen peroxide content

**Figure 4:**
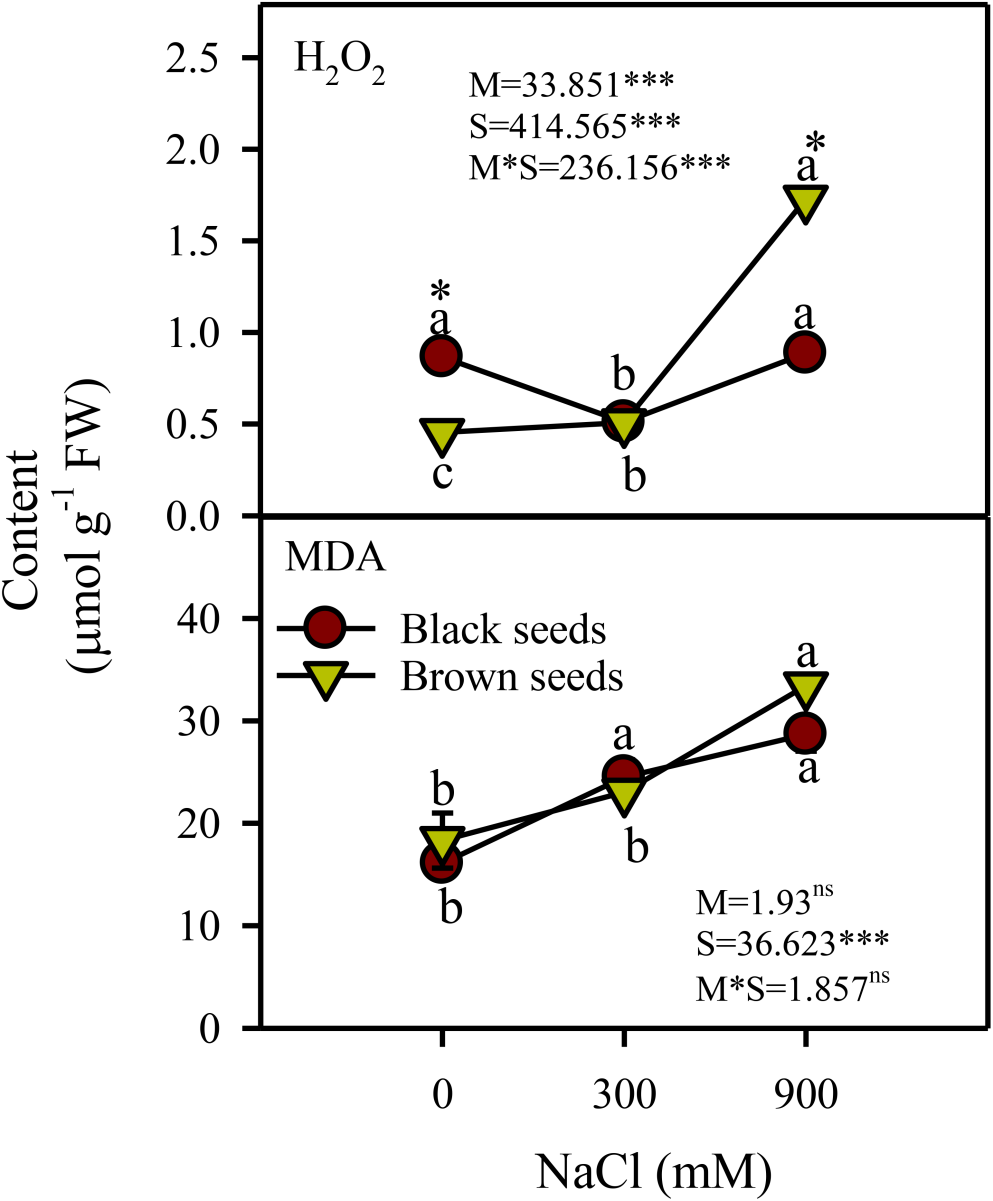
Hydrogen peroxide (H_2_O_2_) and MDA content (µmol g^-1^ FW) in plants obtained from black and brown seed morphs of *A. macrostychum* under different NaCl treatments. Data is given as mean ± standard error. Symbols with different letters are significantly (P <0.05) different from each other (Bonferroni test).

Two-way ANOVA indicated a significant (P<0.001) effect of seed morphology (M), salinity (S), and their interactions (M×S) on hydrogen peroxide content. Hydrogen peroxide (H_2_O_2_) content of plants from black seeds decreased (44%) transiently in moderate salinity compared to control and high salinity treated plants (**Figure 4**). H_2_O_2_ content of plants germinated from brown seeds was unaffected by moderate salinity and showed a 3.5 fold increase under high salinity. Plants derived from brown seeds showed 2 fold higher H_2_O_2_ content under high salinity compared to those from black seeds (**Figure 4**).

### 3.5 Lipid peroxidation

There was a significant (P<0.05) effect of salinity (S) but not of seed morphology (M) and M×S interaction on lipid peroxidation (measured as malondialdehyde) level. In general, lipid peroxidation increased with increasing salinity in plants germinated from either seed type (**Figure 4**).

### 3.6 Enzymatic antioxidants

**Figure 5:**
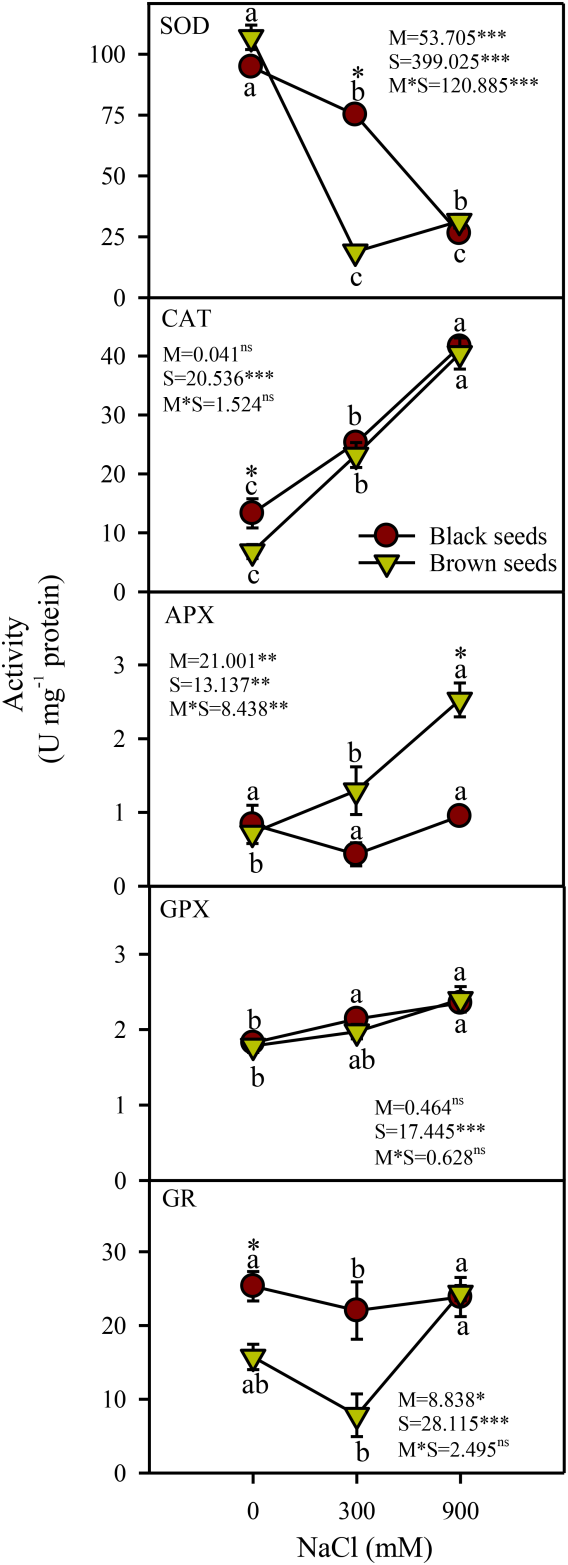
Antioxidant enzyme activities in plants obtained from black and brown seed morphs of *A. macrostychum* under different NaCl treatments. Data is given as mean ± standard error. Symbols with different letters are significantly (P <0.05) different from each other.

Seed morphology (M), salinity (S), and their interactions (M×S) had significant effects on the activity of superoxide dismutase (SOD). SOD activity decreased under saline conditions in plants obtained from either seed type (**Figure 5**). Plants produced from black seeds had 3.8 fold higher SOD activity under moderate salinity compared to those derived from brown seeds. Salinity (S) but not seed morphology (M) and M×S interaction had a significant (P<0.001) effect on the catalase (CAT) and guaiacol peroxidase (GPX) activities. Activities of CAT and GPX from either seed type increased with increases in salinity (**Figure 5**). Activity of ascorbate peroxidase (APX) was affected significantly by seed morphology (P<0.001), salinity (P<0.001), and their interactions (P<0.01). APX activity increased with increases in salinity in plants derived from brown seeds and was unaffected by salinity in plants obtained from black seeds (**Figure 5**). Plants germinated from brown seeds generally had 2.5 fold higher APX activity under saline conditions in comparison to those from black seeds. Activity of glutathione reductase (GR) was affected significantly by seed morphology (P<0.05) and salinity (P<0.001) but not by their interaction. GR activity of plants from black seeds showed a slight (12%) decline under moderate but not high salinity compared to the non-saline control (**Figure 5**). GR activity of plants derived from brown seeds increased in high but not moderate salinity (**Figure 5**).

### 3.7 Non-enzymatic antioxidants

**Figure 6:**
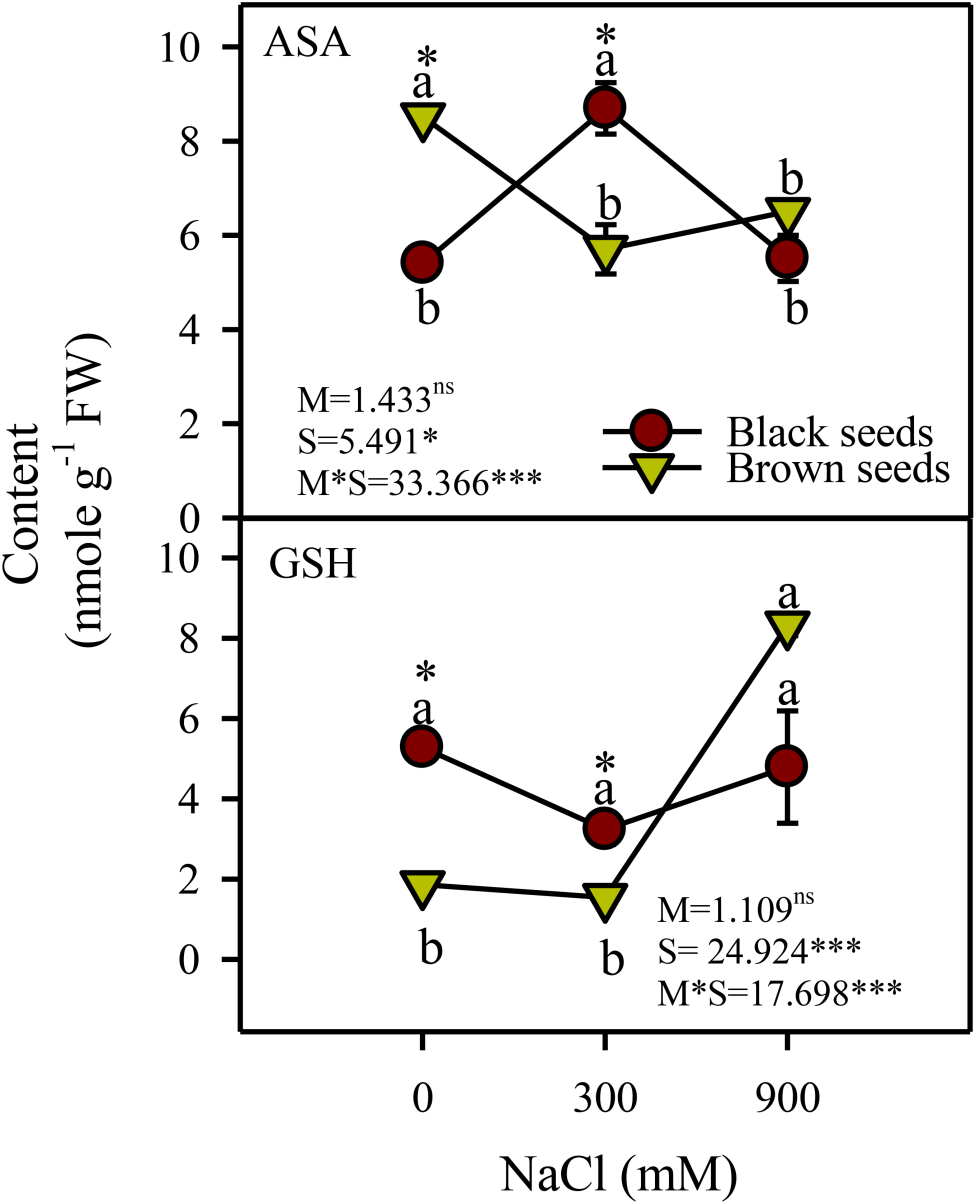
Antioxidant substrates (ascorbate (ASA), and glutathione (GSH) content) in plants obtained from black and brown seed morphs of *A. macrostychum* under different NaCl treatments. Data is given as mean ± standard error. Symbols with different letters are significantly (P <0.05) different from each other (Bonferroni test).

There was a significant effect of salinity (P<0.05) and salinity-seed morphology interaction on the reduced forms of ascorbate (AsA) and glutathione (GSH). AsA content of plants germinated from black seeds increased (1.8 fold) transiently under moderate salinity compared to control and high salinity treatments (**Figure 6**). AsA content of plants from brown seeds decreased under saline conditions. GSH content of plants obtained from black seeds did not vary with salinity and those of plants from brown seeds increased (4.2 fold) only under high salinity (**Figure 6**).

## 4 Discussion

### 4.1 Growth and physio-chemistry under non-saline conditions

Plants of *A. macrostachyum* grown from the two types of heteromorphic seeds generally showed comparable growth (except DW and shoot succulence), sap osmotic potential, activities of most antioxidant enzymes (except CAT and GR), GSH and malondialdehyde content in the absence of salinity. Plants emerged from heteromorphic seeds of *Atriplex centralasiatica* (Xu et al., 2011), *Chenopodium album* (Yao et al., 2010), and *Suaeda aralocaspica* (Cao et al., 2015; Wang et al., 2017) showed similar growth in the absence of salinity. In contrast, *Suaeda splendens* plants grown from heteromorphic seeds showed differences in growth and most physio-chemical parameters under non-saline conditions (Redondo-Gómez et al., 2008). Growth of *Arthrocnemum indicum* plants derived from heteromorphic seeds differed except that sap osmotic potential, MDA content, and activities of most antioxidant enzymes were comparable (Nisar et al., 2021). In this study, *A. macrostachyum* plants from heteromorphic seeds also have some differences in physio-chemical parameters. For instance, plants derived from black seeds showed higher DW, H_2_O_2_, CAT, and GR levels and lower shoot succulence, Chl *a*, CAR, and AsA levels compared to plants germinated from brown seeds under non-saline conditions. Similarly, *A. indicum* plants grown from heteromorphic seeds had differences in FW, shoot succulence, H_2_O_2_ content, AsA, and GSH levels in the absence of salinity (Nisar et al., 2021). Hence, plants from heteromorphic seeds may exhibit commonalities and differences even in the absence of salinity.

### 4.2 Growth and physio-chemistry under moderate salinity

The biomass of *A. macrostachyum* plants grown from heteromorphic seeds under moderate (300 mM NaCl) salinity was comparable to non-saline controls. Similarly, Redondo-Gómez et al. (2010) also reported unaltered shoot biomass fraction and shoot area under moderate (340 mM NaCl) salinity for *A. macrostachyum* from Odiel Marshes, Spain. Biomass of another C_3_ salicornioideae succulent *Halopeplis perfoliata* was also similar to the control when grown in 300 mM NaCl (Rasool et al., 2021). Hence, moderate salinity does not appear deleterious for the growth of salicornioideae halophytes. In this study, *A. macrostachyum* plants from brown seeds had higher shoot FW and plants derived from black seeds had higher root biomass compared to their counterparts under moderate salinity. Similarly, plants of *A. indicum* (Nisar et al., 2021), *Chenopodium album* (Yao et al., 2010), and *Suaeda splendens* (Redondo-Gómez et al., 2008) from heteromorphic seeds showed differences in growth under moderate salinity. In contrast, plants from heteromorphic seeds of the annual halophyte *Suaeda aralocaspica* did not show differential biomass accumulation (Cao et al., 2015; Wang et al., 2017). Hence, growth responses of plants developed from heteromorphic seeds under moderately saline conditions may vary among species.

Decrease in sap Ψ_s_ is a commonly used indicator of osmotic adjustment, which is important for salinity tolerance in plants (Flowers and Colmer, 2008; Hameed and Khan, 2011; Katschnig et al., 2013). In this study, *A. macrostachyum* plants that emerged from heteromorphic seeds displayed a similar decrease in sap Ψ_s_ under moderate salinity compared to the non-saline control with lower values in shoots compared to roots. Plants of *A. indicum* grown from heteromorphic seeds also showed a similar decline in sap Ψ_s_ in 300 mM NaCl compared to the control (Nisar et al., 2021). Likewise, many other halophytes such as *Suaeda maritima* (Moghaieb et al., 2004), *Salicornia dolichostachya* (Katschnig et al., 2013), and *S. europaea* (Moghaieb et al., 2004) also showed a decrease in sap Ψ_s_ under moderate salinity. Root water content and shoot succulence of *A. macrostachyum* plants from heteromorphic seeds remained unchanged under moderate salinity compared to the control, indicating effective osmotic adjustment.

Moderate salinity did not affect Chl *a* levels of plants derived from heteromorphic seeds. Redondo-Gómez et al. (2010) however, reported a decline in Chl *a*, improved midday Fv/Fm, and unchanged ɸPSII under moderate salinity compared to controls in *A. macrostachyum* plants, which could be ascribed to differences in genetic background and maternal environment (Weinhold, 2018; Roman et al., 2020; Araus et al., 2021). However, many other halophytes such as *Salvadora persica* (Rangani et al., 2016) and *Atriplex portulacoides* (Redondo-Gómez et al., 2007) also showed generally unaltered Chl *a* under moderate salinity. Unaffected Chl *a*, in aforementioned halophytes including our test species indicate the resilience of light harvesting machinery to moderate salinity, which could support the maintenance of biomass. However, Chl *b* and CAR increased in plants germinated from black seeds; whereas these parameters remained unaffected by moderate salinity in plants from brown seeds. The Chl *b* and CAR molecules are mainly found in light-harvesting complexes (LHCs; Taiz et al., 2015). In addition, CAR also acts as a protective compound for the LHCs. Hence, a decrease in these compounds, especially Chl *b*, may result in structural/conformational changes in the PSII antennae (Kocheva et al., 2004; Taiz et al., 2015). Although mostly related to Chl *a*, a decrease in Fv/Fm coincided with Chl *b* in leaves of rice under NaCl treatment (Lutts et al., 1996). Hence, increased or unaltered levels of Chl *b* and CAR under moderate salinity in this study might be an adaptation of *A. macrostachyum* plants to maintain the photochemical efficiency of photosynthesis. However, information about light harvesting parameters in plants derived from heteromorphic seeds is non-existent and warrants more studies.

In this study, levels of H_2_O_2_ (i.e. a common ROS) either decreased or remained unchanged in *A. macrostachyum* plants from heteromorphic seeds under moderate salinity, Consequently, a decline in the activity of SOD, which converts superoxide radicles to H_2_O_2_, was observed in plants of test species derived from either type of seeds. Levels of most H_2_O_2_-detoxifying enzymes (except GR in plants from black seeds) and antioxidants (except ascorbate in plants from brown seeds) in plants obtained from heteromorphic seeds were either higher or comparable to non-saline controls. Similarly, levels of most antioxidant enzymes and substances either increased or remained unaltered under moderate salinity in many other salicornioideae halophytes such as *Salicornia brachiata* (except SOD and AsA; Parida and Jha, 2010), *S. persica* and *S. europaea* (Aghaleh et al., 2011). However, there was a 1.2-1.4-fold increase in MDA (an indicator of oxidative membrane damage) under moderate salinity in plants from black and brown seeds, respectively. This rise in MDA might be ascribed to photorespiratory H_2_O_2_, which is a characteristic of C_3_ species (Taiz et al., 2015; Turkan et al., 2018) and 2 to 5-fold increase in CAT activity (in presence of unaltered/lower ETR and decreased SOD) in our test species could be an indicator of photorespiration. Activity of CAT also increased under saline conditions in many other C_3_ halophytes such as *Halopeplis perfolaita* (Rasool et al., 2019) and *Salvadora persica* (Rangani et al., 2016). However, more detailed studies are required in this regard.

### 4.3 Growth and physio-chemistry under high salinity

Plants of *A. macrostachyum* survived high salinity (900 mM NaCl), which was equivalent to ~1.5-fold seawater salinity. Redondo-Gómez et al. (2010) also reported high (1030 mM NaCl) salinity tolerance of *A. macrostachyum* in an earlier study. Similarly, many other salicornioideae halophytes such as *Sarcoconia fruticosa* (1030 mM NaCl; Redondo-Gómez et al., 2006), *A. indicum* (900 mM NaCl; Nisar et al., 2021), *Halopeplis perfoliata* (600 mM NaCl; Rasool et al., 2019), *Salicornia persica* (600 mM NaCl; Aghaleh et al., 2009) and *S. europaea* (600 mM NaCl; Aghaleh et al., 2009) could also tolerate seawater or higher salinity. However, high salinity (900 mM NaCl) caused a comparable decrease in most growth parameters of the *A. macrostachyum* plants developed from heteromorphic seeds. High salinity also caused inhibition of growth in many salicornioideae halophytes, namely *Salicornia europaea* (≥800 mM NaCl; Lv et al., 2012), *Halosarcia pergranulata* (≥600 mM NaCl; Short and Colmer, 1999) and three *Tecticornia* spp. (≥900 mM NaCl; Moir-Barnetson et al., 2016). Decreased growth of halophytes under high salinity could be an adaptive strategy to increase chances of survival long enough to produce some seeds (Neumann, 2011; Hameed et al., 2012).

High salinity (900 mM NaCl; equivalent to about -4MPa Ψ_s_) resulted in a significant (P<0.05) decline in sap Ψ_s_ of shoots (about -7MPa) and roots (about -5MPa) of *A. macrostachyum* plants emerged from heteromorphic seeds, which appears adequate for osmotic adjustment. Likewise, plants of *A. indicum* produced from heteromorphic seeds also showed an osmoconformer response, as sap Ψ_s_ decreased with increases in salinity (Nisar et al., 2021). Hence, plants developing from heteromorphic seeds appear to respond similarly to the osmotic constraint of salinity. Furthermore, plants of *A. macrostachyum* from heteromorphic seeds showed similar shoot succulence and water content of root, which was largely insensitive to high salinity, except for a slight decline in shoot succulence of plants obtained from brown seeds. This finding also hints at effective osmotic adjustment in our test species under high salinity.

Plants of *A. macrostachyum* derived from black seeds had higher Chl *a*, *b*, and CAR under 900 mM NaCl salinity compared to the control, whereas those from brown seeds remained unaffected by high salinity. In contrast, Redondo-Gómez et al. (2010) in an earlier study reported a decline in Chl *a*, *b*, and CAR in *A. macrostachyum* under high salinity. Chlorophyll *a* and *b* contents of congener *A. indicum* in 500 mM NaCl were comparable to the non-saline control (Nagarajan et al., 2008). Likewise, levels of photosynthetic pigments in *Salicornia brachiata* under 500 mM NaCl also remained generally similar to the control (Siddiqui et al., 2022). However plants of *A. macrostachyum* from heteromorphic seeds showed a 72-77% decline in SOD activity under high salinity compared to the control; which could be an indicator of the low incidence of superoxide production through electron leakage to oxygen from ferredoxin at photosystem-I level (Asada, 1999). Furthermore, the level of H_2_O_2_ in plants of *A. macrostachyum* derived from black seeds was comparable to the control. However, H_2_O_2_ content of plants germinated from brown seeds showed a 3.5-fold increase under high salinity in comparison to the control, which could possibly result from photorespiration, which is a characteristic of C_3_ plants (Taiz et al., 2015). The CAT activity in plants from black and brown seeds showed a 3.3 and 8-fold increase under high salinity, respectively. Higher induction of CAT activity in plants obtained from brown compared to black seeds hints at a greater extent of photorespiration-based H_2_O_2_ production in plants germinated from brown seeds. The level of MDA (an indicator of oxidative membrane damage) increased under high salinity in plants from either seed type, but plants germinated from brown seeds had 1.1-fold higher MDA compared to those from black seeds. Activities of all H_2_O_2_-detoxifying enzymes and GSH increased in plants derived from brown but not black seeds, indicating the greater need for H_2_O_2_ detoxification in the aforementioned plants under high salinity. However, this induction was not adequate and plants germinated from brown seeds developed comparatively higher MDA than those from black seeds. Differences in antioxidant defense and levels of MDA were also found in plants grown from heteromorphic seeds of *A. indicum* (Nisar et al., 2021). However, more studies are needed for a better understanding about the differences in antioxidant systems in plants produced from heteromorphic seeds.

## 5 Conclusions

Our data indicates many similarities and differences in growth and physio-chemical responses of plants of *A. macrostachyum* derived from heteromorphic seeds. Moderate salinity (300 mM NaCl) did not cause inhibition of growth or Chl *a* in both types of plants. However, high salinity (900 mM NaCl) led to a decrease in growth and sap ψ_s_ in plants derived from heteromorphic seeds. Decreased SOD levels hint at a low incidence of chloroplastic H_2_O_2_ formation under salinity. However, an increase in MDA levels and CAT activity in plants from both types of seeds suggest extra-chloroplastic H_2_O_2_ generation under increasing salinity. Under high salinity, activities of all H_2_O_2_-detoxifying enzymes and GSH increased in plants obtained from brown but not black seeds; indicating the greater need for H_2_O_2_ detoxification in plants from brown seeds under high salinity. However, this induction was not adequate enough and plants germinated from brown seeds developed comparatively higher MDA than those from the black seeds. Hence, plants derived from black seeds appear to be more resistant to high salinity- induced oxidative damages than those developed from brown seeds. These data indicate metabolic flexibility under increasing salinity in plants of *A. macrostachyum* emerging from heteromorphic seeds. Detailed studies are needed to determine the molecular basis of these similarities and differences.

## Data Availability

The original contributions presented in the study are included in the article/supplementary material. Further inquiries can be directed to the corresponding authors.
